# Dynamic Repositioning of Aerial Base Stations for Enhanced User Experience in 5G and Beyond

**DOI:** 10.3390/s23167098

**Published:** 2023-08-11

**Authors:** Shams Ur Rahman, Ajmal Khan, Muhammad Usman, Muhammad Bilal, You-Ze Cho, Hesham El-Sayed

**Affiliations:** 1 Department of Computer Science, UET Mardan, Charsadda Road, Mardan 23200, Pakistan; shams@uetmardan.edu.pk (S.U.R.); usman@uetmardan.edu.pk (M.U.); 2 Department of Artificial Intelligence and Big Data, Woosong University, 171 Dongdaejeon-ro, Dong-gu, Daejeon 34606, Republic of Korea; engajmal@wsu.ac.kr; 3 Department of Computer Engineering, Hankuk University of Foreign Studies, Yongin-si 17035, Republic of Korea; m.bilal@ieee.org; 4 School of Electronics and Electrical Engineering, Kyungpook National University, Daegu 41566, Republic of Korea; 5 Computer and Network Engineering Department, College of Information Technology, United Arab Emirates University, Al Ain 16427, United Arab Emirates; helsayed@uaeu.ac.ae

**Keywords:** 5G, dynamic repositioning, ultra-dense deployment, UAV network, throughput maximization, optimal positioning

## Abstract

The ultra-dense deployment (UDD) of small cells in 5G and beyond to enhance capacity and data rate is promising, but since user densities continually change, the static deployment of small cells can lead to wastes of capital, the underutilization of resources, and user dissatisfaction. This work proposes the use of Aerial Base Stations (ABSs) wherein small cells are mounted on Unmanned Aerial Vehicles (UAVs), which can be deployed to a set of candidate locations. Furthermore, based on the current user densities, this work studies the optimal placement of the ABSs, at a subset of potential candidate positions, to maximize the total received power and signal-to-interference ratio. The problems of the optimal placement for increasing received power and signal-to-interference ratio are formulated, and optimal placement solutions are designed. The proposed solutions compute the optimal candidate locations for the ABSs based on the current user densities. When the user densities change significantly, the proposed solutions can be re-executed to re-compute the optimal candidate locations for the ABSs, and hence the ABSs can be moved to their new candidate locations. Simulation results show that a 22% or more increase in the total received power can be achieved through the optimal placement of the Aerial BSs and that more than 60% users have more than 80% chance to have their individual received power increased.

## 1. Introduction

5G networks and beyond face the enormous challenge of satisfying the ever-increasing demand for more user capacity, higher data rates, and lower latency. These demands are fueled by the proliferation of increasingly data-hungry smart devices and applications such as augmented reality, virtual reality, and 3D multimedia. Ericsson Mobility Report 2022 [1] projections show that by the end of 2022, monthly average usage per smartphone will surpass 15 GB, and by 2027, 5G subscriptions will reach 4.4 billion. Moreover, according to the report, traffic has grown by more than 15 times in the past 5 years (as of June 2022), and the total mobile traffic will surge to 328 Exabytes per month in 2028, which will be a 5× increase compared to 2022. According to the report, 5G will be the main driver for fueling these demands. To meet such demands, 5G aims to bring about a 100× increase in capacity and a 1000× increase in user data rates compared to 4G [2]. To achieve these ambitious targets, a variety of advanced techniques are being employed, including massive MIMO (Multiple-Input, Multiple-Output), millimeter Wave, and ultra-dense deployment (UDD) of small cells [2].

**Figure 1:**
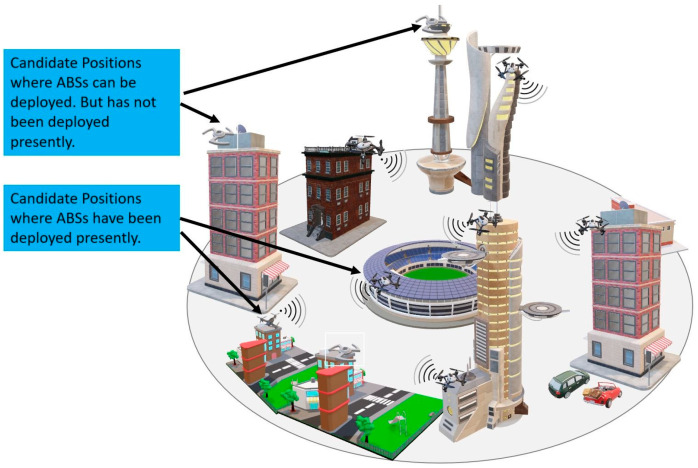
Aerial Base Station-based ultra-dense mobile network.

UDD involves the deployment of many closely spaced base stations which incurs tremendous capital expenditure (CAPEX) and operational expenditure (OPEX); therefore, it is important to maximize the utilization of UDD resources. However, due to user mobility and activities, user density changes all the time [3]. For example, user density around shopping areas is higher on weekends and in the evening compared to weekdays and work hours, respectively; in the evening time, user density around parks is higher compared to work hours; during sports events, density around sports venues is drastically higher; and during rallies and protests, user density constantly changes. Such fluctuations in user density result in some parts of the network being overloaded, whereas other parts are under-utilized if the deployment is fixed. Therefore, it is highly desirable, on the part of service providers, to be able to adjust the deployment, i.e., location, of small cells according to user density, which can be achieved by mounting the small cells onto unmanned aerial vehicles (UAVs), which we will call Aerial Base Stations (ABSs), as shown in Figure 1. The use of such an ABS-based network offers an attractive solution to the problem of network under-utilization due to varying user density and provides the service provider with a significant reduction in CAPEX and OPEX.

In the deployment of an ABS-based network, an immediate problem that arises is the optimal placement of the ABSs based on the user density distribution. Since UDNs are aimed at increasing capacity while ensuring the best user experience, such as providing higher data rates anywhere, therefore, for enhanced spectral efficiency, the received power and signal-to-interference ratio (SIR) should be higher. In this work, we study the problem of the optimal placement of ABSs at a subset of candidate locations that maximizes the total received power and the total SIR. A candidate position is a location where an ABS can be deployed and perched on a platform while it is deployed there. The deployed ABS can receive power at that location; so, when an ABS reaches its deployed candidate location, it does not need to hover. Furthermore, it means that there is no risk of a UAV or small cell running out of energy while it is operating at a candidate location. To the best of our knowledge, no other work has studied the problem of the optimal placement of ABSs at a set of discrete candidate locations as described above.

The main contributions of this work are as follows:

- This paper proposes a novel idea of potential candidate positions, a subset of which will be chosen for the placement of ABSs based on user density.
- This paper formulates the problem of optimal ABS placement at a subset of the candidate positions to maximize received power and SIR.
- This paper develops solutions for the formulated problems to determine the optimal placement of ABSs.

The rest of the paper is organized as follows. Section 2 gives a brief overview of related works. Section 3 describes the system model. In Section 4, the problem is formulated, whereas in Section 5, a solution for the problem is modeled in MATLAB. Section 6 discusses the simulation results and Section 7 draws some conclusions from this work.

## 2. Related Works

In recent years, researchers have been actively investigating the use of UAVs for providing communication services in a variety of scenarios, such as disaster-hit scenarios [4,5], extending coverage of fixed infrastructure [6], dealing with network overload conditions [7], etc. Moreover, the use of UAVs for enhancing the capabilities of the 5G network has also been investigated [8]. This work considers a UAV-based small cell network and investigates how ABSs can be optimally placed based on user density for improving received power and SIR.

The placement of ABSs affects various network performance parameters, such as coverage, throughput, connectivity, and revenue. The issue of mobile base station placement to maximize coverage is also studied in Wireless Sensor Networks (WSNs), where the goal is to place the base stations in a way that maximizes the number sensor nodes being covered, which resembles the problem of coverage maximization in mobile networks. There are works that attempt to maximize throughput or spectrum efficiency. In [9], the authors proposed an algorithm for optimizing the position of a single UAV for throughput maximization of users based on the user data rate requirements and their positions. In [4] the authors proposed an algorithm that determines optimal positions of multiple ABSs to maximize throughput considering a software-defined wireless network deployed in a disaster-hit area. In [10], the authors used deep learning to determine optimal UAV positions to maximize throughput. The authors of [11] studied maximizing the sum rate via power control and UAV positioning. Rosario et al. [12] studied the problem of UAV-based relays’ placement to support high-quality live video transmission. In [13], the authors investigated the optimal altitude of low-altitude aerial platforms for coverage maximization.

Other works exploit the dynamic nature of UAV positioning to enhance network capacity. For example, considering a multi-tier drone-cell network to complement a terrestrial heterogeneous network, BorYaliniz et al. [14] studied the positioning of drone base stations (BSs) to maximize coverage and revenue. Guo et al. [15] conducted a theoretical analysis of how interference affects capacity and worked out a closed-form expression determining optimal ABS locations. Kalantari et al. [16] investigated the three-dimensional (3D) placement of ABSs to maximize the number of served users and their total rate. A mobility control algorithm has been proposed by Dixon et al. [17] for the optimal placement of a chain of UAV-based communication relays for enabling end-to-end communication. In [18], the authors investigated the optimum altitude for both static and mobile UAVs and its effects on the total power loss and bit error rates, etc. The authors of [19] propose an algorithm for the 3D placement of ABSs to maximize the coverage of users with different quality of service (QoS) requirements.

Since fixed-winged UAVs cannot hover at a particular location, there are works that attempt to enhance various network parameters through trajectory control of the UAVs. For example, Cheng et al. [20] proposed an iterative algorithm to determine optimal UAV trajectory for offloading traffic at the edge regions of three adjacent base stations. Zhan et al. [21] proposed an algorithm aimed at optimizing the performance of a ground-to-relay link by controlling the heading angle of the UAV. None of the solutions, and, to the best of our knowledge, any other related works, are suitable to efficiently solve the problem of receiving power and SIR maximization in ABS-based UDNs. Hence, this work studies this problem and designs a solution for it.

## 3. System Model

Let *U* = {1,2,3, …, *N_u_*} be the set of ABSs and *S* = {1, 2,3, …,*N_s_*} be the set of users. Let *L* = {1,2,3, …,*N_l_*} be the set of *N_l_* candidate locations for UAV placement. There are more candidate locations than the number of UAVs, i.e., *N_l_* > *N_u_*. All ABSs have the same transmission power *P_s_*. We assume that the main source of noise for a user is the interference caused by ABSs other than the one with which the user is associated and that the thermal noise is negligible.

### 3.1. Aerial Base Stations

The small cells are mounted on UAVs called ABSs, and their deployment locations can be changed in response to user changes in density and mobility. We assume that the ABSs can autonomously move to the candidate location, perch, and connect to the power source there. Exactly how these tasks will be accomplished is outside the scope of this work, but given the rapid advancements in the autonomous flight of UAVs and localization, these assumptions are realistic.

### 3.2. Candidate Locations for ABSs

A candidate position is a location where an ABS can be deployed. We assume that each candidate position consists of a platform where an ABS can perch while it is deployed there and that the ABS can receive power at that location; so, when an ABS reaches its deployed candidate location, it does not need to hover. Furthermore, it means that there is no risk of a UAV or small cell running out of energy while it is operating at a candidate location. Furthermore, the batteries of a UAV can be charged while it is perched and operating at the candidate location. We assume that each candidate position has sufficient backhaul capacity to support the maximum possible traffic load generated at an ABS.

### 3.3. Path Loss Model

The air-to-ground path loss model [13,22] is used, which gives, in a probabilistic manner, the average path loss:

(1) $$PL\left(dB\right)=20\log\frac{4\pi \;f_{c}d}{c}+P(NLoS)\eta_{NLoS}$$

where *d* is the distance between the ABS and receiver, *P*(*LoS*) is the line-of-sight (LoS) probability, and *P*(*NLoS*) is the non-line-of-sight probability. Furthermore, *η_LoS_* and *η_NLoS_* represent the additional losses in the cases of LoS and NLoS, respectively. The value for *d* is given by $\sqrt{h^{2}+r^{2}}$, where *h* and *r* are the altitudes of the UAV and its horizontal distance from the receiver, respectively. *P*(*LoS*) is given by

(2) $$P(LoS)=\frac{1}{1+a\;\exp(-b(\theta -a))}$$

where *a* and *b* are constants depending on the environment and *θ* is the elevation angle (in degrees) given by $\frac{180}{\pi }\arctan\left(\frac{h}{r}\right)P\left(NLoS\right)=1-P(LoS)$.

## 4. Problem Formulation

Let *A_i_* be a zero–one indicator variable with *A_i_* = 1 if an ABS is deployed at candidate location *i;* otherwise, *A_i_* = 0. Similarly, let *B*_*i*,*j*_ be a zero–one indicator variable with *B*_*i*,*j*_ = 1 if user *j* is connected to the ABS at candidate location *i*; otherwise, *B*_*i*,*j*_ = 0. Let *P*_*i*,*j*_ be the power received by user *j* from the ABS at location *i*. If user *j* is connected to the ABS at location *r*, then the signal-to-interference noise ratio at user *j* will be:

(3) $${\mathrm{SIR}}_{j}=\frac{P_{r,j}}{\sum_{i\in L,i\neq r}P_{i,j}}$$

The goal is to place the ABSs in candidate locations and to assign users to ABSs to maximize the sum of SIR for all users, that is,

(4) $$\underset{A_{i},B_{i,j},\forall i\in L,\forall j\in S}{\mathrm{maximize}}\sum_{j\in S}{\mathrm{SIR}}_{j}$$

If all the ABSs use different frequency bands, then the goal will be to maximize the sum of received powers (i.e., received signal strength (RSSI)). Let *P_j_* be the power received by a user *j* from the candidate location to which it is assigned; then,

(5) $$\underset{A_{i},B_{i,j},\forall i\in L,\forall j\in S}{\mathrm{maximize}}\sum_{j\in S}P_{j}$$

In Equations (4) and (5), *A_i_* controls where to place an ABS and *B*_*i*,*j*_ controls to which location to assign a user such that the corresponding summation is maximized.

A user *j* can only be assigned to a candidate location *i* if there is an ABS placed at that candidate location, i.e., *A_i_* = 1. This means:

(6) $$A_{i}B_{i,j}=1,\forall j\in S$$

A user should be assigned to exactly one candidate location, which means:

(7) $$\sum_{i\in L}B_{i,j}=1,\forall j\;\in \;S$$

As *N_u_* is the number of ABSs, the following constraint should be satisfied:

(8) $$\sum_{i\in L}A_{i}=N_{u}$$

### 4.1. Maximizing the Sum of Received Powers

The problem of maximizing the sum of powers is formally defined below.

(9a) $$\underset{A_{i},B_{i,j},\forall i\in L,\forall j\in S}{\mathrm{maximize}}\sum_{j\in S}P_{j}$$

subject to: *A_i_B*_*i*,*j*_ = 1,∀*j* ∈ *S* (9b)

(9c) $$\sum_{i\in L}Bi,j$$

(9d) $$\sum_{i\in L}A_{i}$$

### 4.2. Maximizing the Sum of SIRs

The problem of maximizing the sum of SIRs is formally defined below.

(10a) $$\underset{A_{i},B_{i,j},\forall i\in L,\forall j\in S}{\mathrm{maximize}}\sum_{j\in S}{\mathrm{SIR}}_{j}$$

subject to: *A_i_B*_*i*,*j*_ = 1, ∀*j* ∈ *S* (10b)

(10c) $$\sum_{i\in L}Bi,j$$

(10d) $$\sum_{i\in L}A_{i}$$

## 5. Solution Modeling

The optimization problems posed in Section 4.1 and Section 4.2 are 0–1 integer programming problems and computationally fall in the class of NP-complete problems. We used MATLAB’s bintprog to solve these problems.

The function [X Val] = bintprog (F, A, B, A_eq_, B_eq_) produces a vector X whose entries are binary values and a variable Val = F^T^X, which is the value that is minimized; F^T^ represents the transpose of F. The arguments A_eq_ and B_eq_ are used for equality constraints such that A_eq_X = B_eq_, whereas A and B are used for inequality constraints such that AX ≤ B. A_eq_ is a matrix whose number of rows should equal the number of equality constraints and whose columns should equal to the number of 0–1 variables.

Based on the positions of users and candidate locations, applicable channel model, transmission power, etc., for each user, the RSSI from each candidate position is calculated and stored in an *N_s_*-by-*N_l_* matrix P, where *P*_i,j_ represents the power received by user *i* from candidate location *j*.

For our problem of received power maximization defined in Section 4.1, A_eq_ is an (*N_s_* + 1)-by-(*N_s_* + 1)*N_l_* matrix, whereas B_eq_ is an (*N_s_* + 1)-by-1 vector. This means that there are *N_s_* + 1 equality constraints. The first equality constraint ensures that exactly *N_u_* UAVs (small cells are deployed and the remaining *N_s_* constraints ensure that each user is assigned to exactly one candidate location. (Note that we ensure through inequality constraints that each user is assigned to a candidate location with a deployed small cell; we will discuss this shortly). F is an (*N_s_* + 1)*N_l_*-by-1 vector and is constructed such that the first *N_l_* entries are set to zero and entries *iN_l_* + 1 through (*i* + 1)*N_l_* are set to the received powers of user *i* from all candidate locations: 1 through *N_l_*. A_eq_ is constructed as given in Algorithm 1.
**Algorithm 1:** Construction of matrix A_eq_ for received power maximization**Input:** Number of users, *N_s_* and Number of candidate positions, *N_l_***Output:** Matrix A_eq_**for** *i* = 1 **upto** *N_s_* + 1  A_eq_[*i*][(*i* − 1)*N_l_* + 1:(*i* − 1) *N_l_* + *N_l_*] = 1 **end**

The vector B_eq_ is constructed as B_eq_ = [*N_u_* O]^T^, where *N_u_* is the number of ABSs and O is row vector of size N_s_ and each of its entries is 1. Moreover, A is an *N_s_N_l_* by (*N_s_* + 1)*N_l_* matrix, whereas b is an *N_s_N_l-_*by-1 vector. Then, for the inequality constraint, the matrix is constructed as given in Algorithm 2.
**Algorithm 2:** Construction of matrix A for received power maximization**Input:** Number of users, *N_s_* and Number of candidate positions, *N_l_***Output:** Matrix A**for** *i* = 1 **upto** *N_s_* **for** *j* = 1 **upto** *N_l_*  A[(*i* − 1)*N_l_* + *j*][*j*]=1  A[(*i* − 1)*N_l_* + *j*][*iN_l_* + *j*] = −1 **end** **end**

B is an *N_s_N_l_*-by-1 column vector and is initialized to all zero entries; that is, B[1:*N_s_N_l_*] = 0. The matrix F is constructed as shown in Algorithm 3.
**Algorithm 3:** Construction of vector F for received power maximization**Input:** Number of users, *N_s_*, Number of candidate positions, *N_l_* and Matrix of received powers, P**Output:** Vector FF[1:*N_l_*] = 0 **for** *i* = 1 **upto** *N_s_* F[*iN_s_* + 1: (*i* + 1)*N_s_*] = P[*i*][:] **end** F = F^T^

For the problem of SIR optimization defined in Section 4.2, matrix A_eq_ is constructed as given in Algorithm 4.
**Algorithm 4:** Construction of matrix A_eq_ for SIR maximization**Input:** Number of users, *N_s_* and Number of candidate positions, *N_l_***Output:** Matrix A_eq_Take two auxiliary matrices T and Z, each of dimensions (*N_s_* + 1)-by-(*N_s_ N_l_* + *N_l_*) of all zero entries **for** *i* = 1 **upto** *N_s_* + 1 T[*i*][(*i* − 1)*N_l_* + 1:(*i* − 1) *N_l_* + *N_l_*] = 1 **end** $A_{\mathrm{eq}}=\left[\begin{array}{cc} T & Z\\ Z & T \end{array}\right]$ A_eq_[1][ *N_l_* + 1:2(*N_s_ N_l_* + *N_l_*)] = 0

Then, B_eq_ is constructed as B_eq_ = [*N_u_* O *N_u_* (*N_u_* − 1)O]^T^, where O is a row vector of size *N_s_* containing all ones.

The inequality constraint matrices A for SIR maximization is constructed as shown in Algorithm 5.
**Algorithm 5:** Construction of matrix A for SIR maximization**Input:** Number of users, *N_s_* and Number of candidate positions, *N_l_***Output:** Matrix ATake four auxiliary amatrices A_1_, A_2_, A_3_ and A_4_ each of dimensions *NsNl-*by-(*N_s_* + 1)*N_l_*Initialize A_1_, A_2_, A_3_ and A_4_ to zero (that is, make all their entries zero) **for** *i* = 1 **upto** *N_s_*  **for** *j* = 1 **upto** *N_l_*  A_1_[(*i* − 1)*N_l_* + *j*][*j*] = 1  A_1_ [(*i* − 1)*N_l_* + *j*][*iN_l_* + *j*] = −1  A_2_[(*i* − 1)*N_l_* + *j*][*j*] = 1  A_3_[(*i* − 1)*N_l_*+*j*][*iN_l_* + *j*] = −1   **end** **end** $A=\left[\begin{array}{cc} A_{1} & A_{4}\\ A_{2} & A_{3} \end{array}\right]$

B is a (2*N_s_N_l_* + *N_l_*)-by-1 column vector and is initialized to all zero entries.

The matrix F is then constructed as given in Algorithm 6.
**Algorithm 6:** Construction of vector F for received power maximization**Input:** Number of users, *N_s_*, Number of candidate positions, *N_l_* and Matrix of received powers, P**Output:** Vector FF_1_[1:*N_l_*] = 0; **for** *i* = 1 **upto** *N_s_* F_1_[*iN_s_* + 1: (*i* + 1)*N_s_*] = P[*i*][:] F_1_ = F_1_^T^ $F=\left[\begin{array}{c} F1\\ -F1 \end{array}\right]$

**Figure 2:**
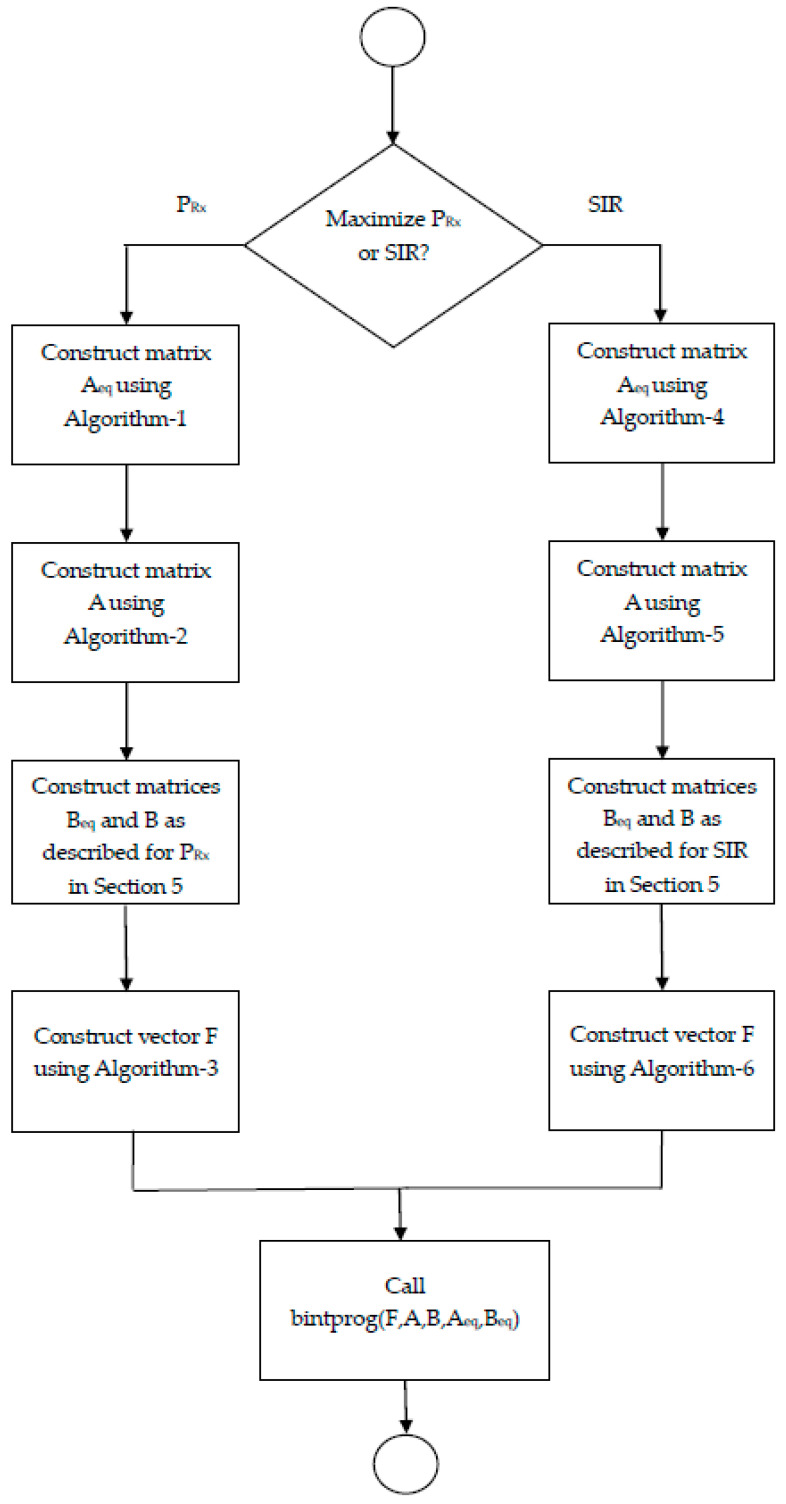
Flowchart indicating the steps the proposed solutions involve.

Figure 2 shows the flowchart of the designed solutions. The flowchart first checks whether the received power or the SIR needs to be maximized. Once this choice is made, the matrices and vectors needed for the bintprog as input are constructed in a series of steps. As described above, this construction ensures that all constraints, as specified in the problem formulation, are satisfied. As evident from the discussion above and from the flowchart, the construction processes for each problem is different from the other and involves using matrices and vectors of certain dimensions and then filling out certain entries in those matrices and vectors, thereby incorporating the problem constraints and ensuring, for each problem, that the bintprog properly evaluates the objective function.

## 6. Results and Discussion

In this section, we discuss the MATLAB simulation results obtained while studying the effect of various parameters on performance improvement due to the optimal placement of the Aerial BSs.

The deployment area was kept at 1000 × 1000 m^2^. The heights of candidate positions (and hence those of the ABSs) were maintained between 50 and 100 m. The transmit power of the ABSs was kept at 30 dBm. User positions were generated using the Poisson point process and Poisson cluster process. A Poisson cluster process is a mathematical model used to describe the spatial distribution of points in a given region. In this process, points are grouped into clusters, where each cluster is generated independently according to a Poisson distribution. The superposition of these clusters allows for individual points to be random and have the tendency to cluster together in certain regions. Candidate positions for ABSs were generated using evenly spaced grid points. A suburban environment was considered with the parameters given in Table 1.

### 6.1. Results

**Figure 3:**
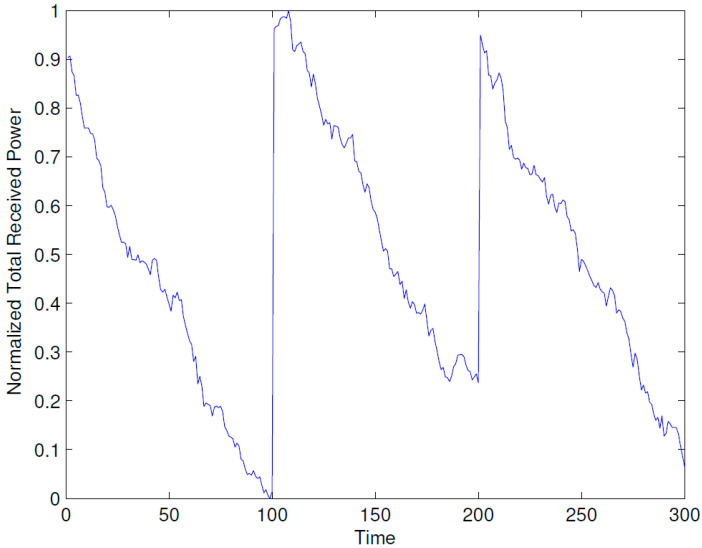
Degradation in total received power due to mobility of users.

Figure 3 demonstrates how the dynamic repositioning of BSs enhances performance. In particular, the figure shows how the optimal placement of the ABSs becomes suboptimal due to the mobility of users. With the passage of time, the suboptimality increases and, hence, the normalized total received power decreases. After a time of 100 epochs, the optimal positions for the ABSs are recomputed, and the ABSs are repositioned, which results again in the maximum achievable total received power. Note that the vertical scale has been normalized between 0 and 1 by subtracting the minimum value from the data and then dividing it by the maximum value.

**Figure 4:**
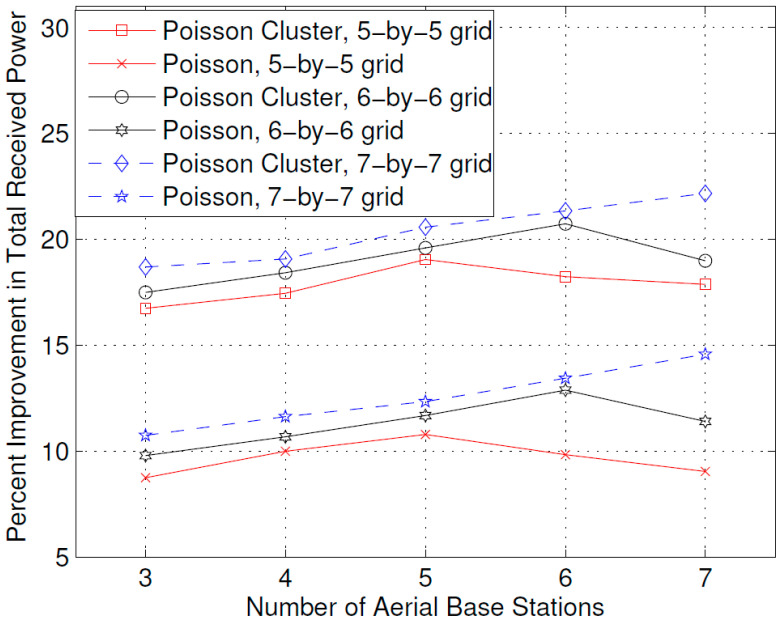
Percent improvement in received power with respect to the number of Aerial BSs.

Figure 4 shows the effect of the number of ABSs on the improvement in the total received power that is achieved with the optimal placement of the ABSs compared to a uniform distributed placement. The user positions were created using two different deployment processes: the Poisson cluster process and the Poisson process. In the Poisson cluster process, users tend to concentrate around certain hot spots. The candidate positions of the ABSs were created using squared grids of 5-by-5, 6-by-6, and 7-by-6. From Figure 4, it is evident that greater improvement in the total received power is achieved with the optimal placement of the ABSs compared to the Poisson cluster process. Moreover, it is evident from the figure that by increasing the grid resolution (number of candidate positions), greater improvement in the total received power is achieved. Furthermore, it can also be observed that maximum improvement is achieved when the number of ABSs is equal to the square root of the grid dimensions: that is *N*, for a grid of *N*-by-*N* maximum improvement is achieved when the number of ABSs is *N*.

**Figure 5:**
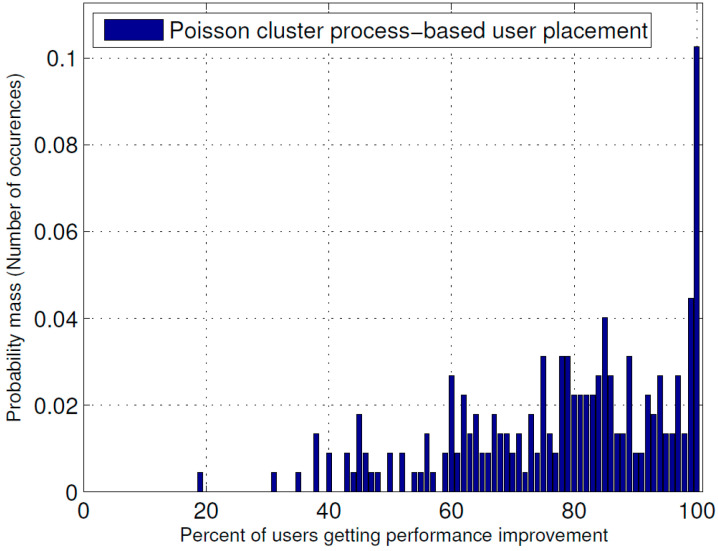
PMF of the percentage of users receiving performance improvement when user positions were generated as Poisson cluster process.

**Figure 6:**
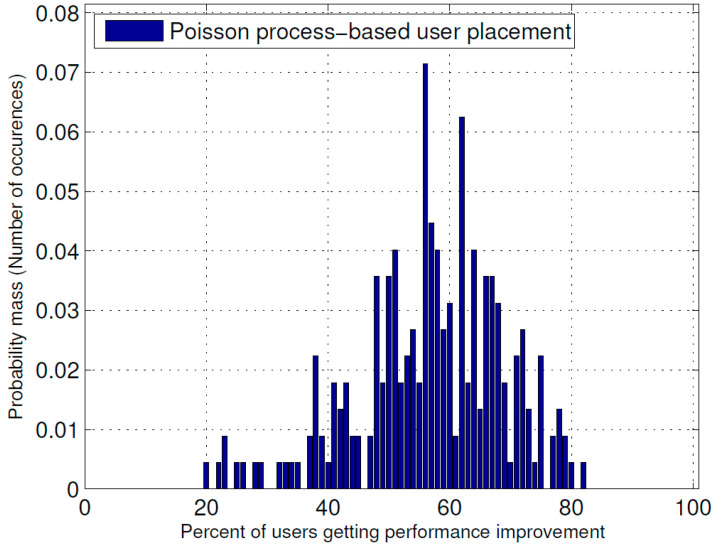
PMF of the percentage of users receiving performance improvement when user positions were generated as Poisson point process.

**Figure 7:**
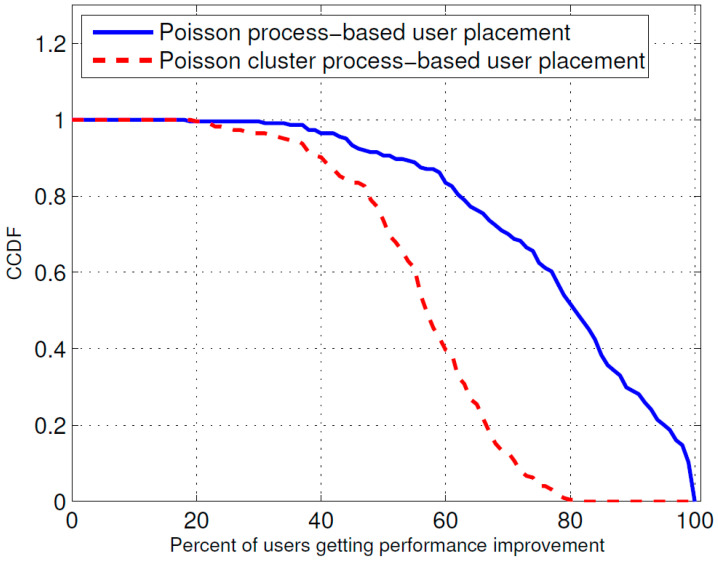
CCDF for percentage of users receiving performance improvement.

Figure 5 and Figure 6 show the probability mass function (PMF) of the percentage of users receiving improvement in their received power due to the optimal placement of the ABSs for the Poisson cluster process and Poisson process of user position generation, respectively, whereas Figure 7 shows their cumulative probability distribution function (CCDF). From these figures, it is evident that in the Poisson cluster process, there is more than a 0.8 probability for 60 percent or more users to receive improvement because of optimal placement of ABSs and more than a 0.5 probability for 80 percent or more users. Similarly, for the Poisson process, there is more than a 0.8 probability for 40 percent or more users to receive improvement due to the optimal ABS placements and an approximately 0.4 probability for 60 percent or more users.

**Figure 8:**
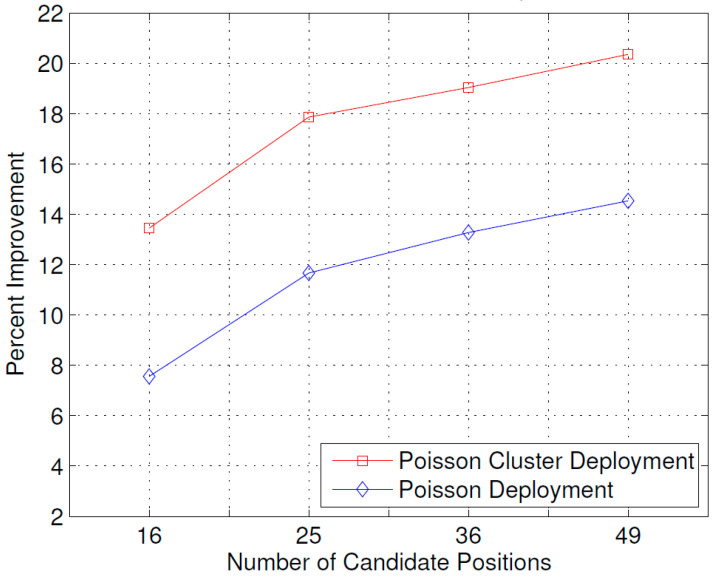
Effect of the number of candidate positions on improvement in received power.

Figure 8 shows the effect of the number of candidate positions on the improvement in received power due to the optimal placement of the ABSs. Increasing the number of candidate positions results in more improvement in the received power. This can be explained by the fact that with more candidate positions, there are more suitable positions for placing the Aerial BSs compared to fewer candidate positions.

**Figure 9:**
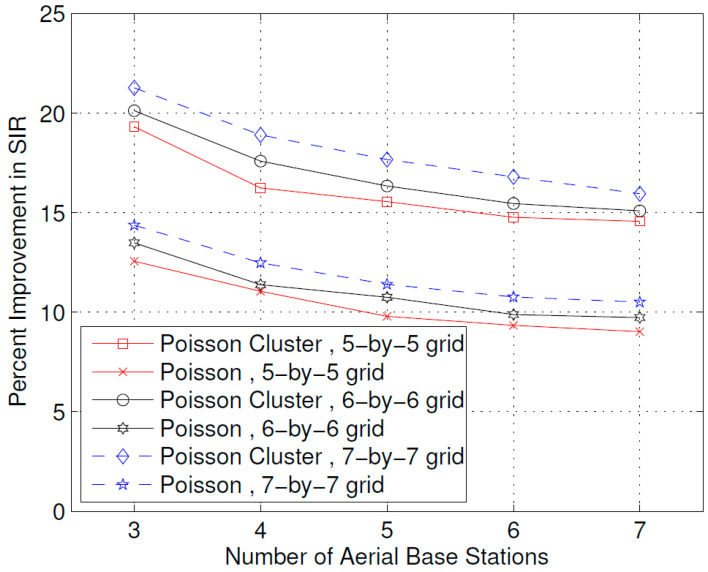
Percent improvement in SIR with respect to number of Aerial BSs.

Figure 9 shows the effect of the number of ABSs on the improvement in the SIR obtained due to optimal placement of the ABSs. It is interesting to note that, unlike the case for received power, as shown in Figure 4, the increase in the number of ABSs leads to a decrease in percent improvement in the SIR. This may be explained by the fact that when the number of ABSs increases, the amount of interference that a user receives also increases, as concluded by some stochastic geometry studies of UDNs [23]; therefore, although the optimal placement still achieves an improvement in the SIR, this improvement is lower compared to the case when there are fewer ABSs. To reduce the effects of increased interference due to the increase in the number of ABSs, one solution can be to assign different channels to neighboring ABSs or use beamforming techniques [24,25]. Channel allocation and beamforming are, however, out of the scope of this work.

**Figure 10:**
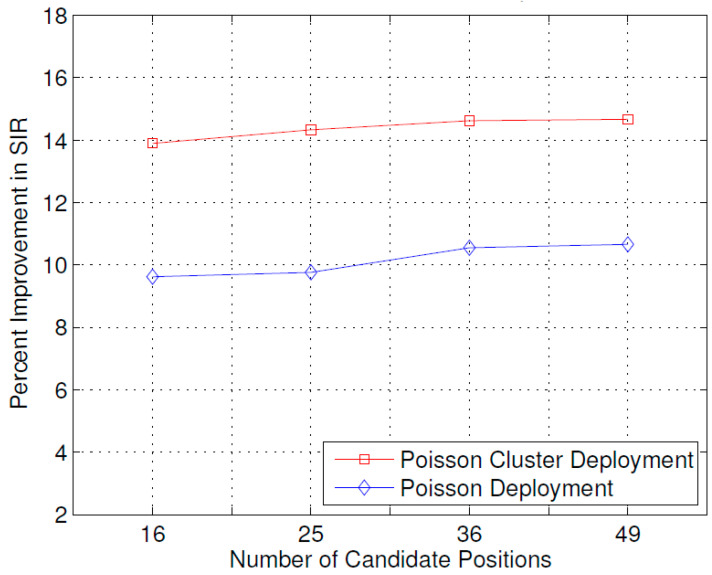
Effect of the number of candidate positions on improvement in SIR.

Figure 10, just like the case for total received power shown in Figure 8, shows the effect of the number of candidate positions on the improvement in SIR achieved by the optimal placement of the ABSs. As the number of candidate positions increases, the improvement in the received SIR also increases for both user distribution types; however, the rate of increase, in this case, is lower compared to that of the total received power, as shown in Figure 8. The same reasons given in explanation of the results of Figure 9 may be attributed to this observation.

### 6.2. Discussion

The results presented above support the idea that the dynamic repositioning of ABSs based on user densities can significantly enhance user experience. For Example, Figure 3 shows that when ABSs are placed at optimal candidate positions, users’ received power is higher, but with the passage of time and user mobility, that placement becomes suboptimal. Therefore, the need for recomputing the optimal positions arises again, and when that is complete and the ABSs are moved to the newly computed optimal candidate positions, the users’ received power significantly increases. Evidently, this dynamic repositioning would not be possible without the use of ABSs, or any other aerial platform that could carry the transmission equipment and move to a target location. Figure 4 shows that depending on the arrangement of the candidate positions, increasing the number of ABSs to a certain limit results in a greater increase in the received power; however, that improvement starts to fall beyond that optimal number of ABSs, which, in the produced results, appears to be the square root of the number of candidate positions. Figure 4, Figure 5 and Figure 6 together indicate that a significant number of users benefit from the dynamic repositioning of ABSs in the form of improved received power and improved SIR. The percentage of users receiving improved received power is higher than the percentage of users receiving improved SIR. This indicates that if neighboring ABSs are allotted different channels, the performance improvement will be greater. Figure 8 shows that increasing the number of candidate positions leads to more improvement in the percentage improvement in the received power. This is because with more candidate positions, there is more flexibility in the placement of the ABSs, which aligns better with the benefits associated with the dynamic placement of ABSs. Figure 9 shows that increasing the number of ABSs leads to more interference and hence less improvement in SIR, which is consistent with other studies on the impact of the number of base stations on the SIR such as [23]. Figure 10 shows that increasing the number of candidate positions leads to greater improvement in the SIR; however, the rate of increase is smaller compared to that of received power, as shown in Figure 8. The overall observation is that the dynamic placement of ABSs has the potential for improving user experience and that the greater the number of candidate positions, the higher the improvement. Moreover, allocating different channels to neighboring ABSs leads to more improvement in performance with dynamic placement.

## 7. Conclusions and Future Work

A novel idea for the dynamic placement (repositioning) of small cells based on user locations and concentrations was proposed. The problem was formally formulated, and an optimization solution was designed and implemented in MATLAB. The effect of various parameters was studied on the improvement in received power and SIR. The results showed that by increasing the number of Aerial BSs, a greater increase in received power can be achieved. Similarly, increasing the number of candidate positions also leads to a greater increase in received power and SIR. Furthermore, when the user positions are more concentrated in hot spots, the optimal placement results in significantly more improvement in the received power and SIR, which range between 16 and 22 percent and 13 and 21 percent, respectively. When the user positions are distributed as the Poisson point process, the improvement in received power ranges between 8 and 15 percent and 7 and 15 percent, respectively. This work studied the improvement in received power and SIR due to the dynamic placement, and in future work, other aspects of dynamic placement may be studied. For example, a future extension of this work might be to study the joint optimization of ABS placement and channel allocation. Another possible extension might be the joint capacity enhancement and user experience optimization.

## Figures and Tables

**Table 1:** Parameters for the suburban environment.

Parameter	Value
a	4.88
b	0.49
*ηLoS*	0.1 dB
*ηNLoS*	21 dB

## Data Availability

Not applicable.
